# Quality of Decision Support in Computerized Provider Order Entry: Systematic Literature Review

**DOI:** 10.2196/medinform.7170

**Published:** 2018-01-24

**Authors:** Delphine Carli, Guillaume Fahrni, Pascal Bonnabry, Christian Lovis

**Affiliations:** ^1^ Division of Pharmacy University Hospitals of Geneva Geneva Switzerland; ^2^ School of Pharmaceutical Sciences University of Geneva, University of Lausanne Geneva Switzerland; ^3^ Division of Medical Information Sciences University Hospitals of Geneva Geneva Switzerland; ^4^ School of Medicine University of Geneva Geneva Switzerland

**Keywords:** decision support systems, clinical, medical order entry systems, system, medication alert, sensitivity, specificity, predictive value of tests

## Abstract

**Background:**

Computerized decision support systems have raised a lot of hopes and expectations in the field of order entry. Although there are numerous studies reporting positive impacts, concerns are increasingly high about alert fatigue and effective impacts of these systems. One of the root causes of fatigue alert reported is the low clinical relevance of these alerts.

**Objective:**

The objective of this systematic review was to assess the reported positive predictive value (PPV), as a proxy to clinical relevance, of decision support systems in computerized provider order entry (CPOE).

**Methods:**

A systematic search of the scientific literature published between February 2009 and March 2015 on CPOE, clinical decision support systems, and the predictive value associated with alert fatigue was conducted using PubMed database. Inclusion criteria were as follows: English language, full text available (free or pay for access), assessed medication, direct or indirect level of predictive value, sensitivity, or specificity. When possible with the information provided, PPV was calculated or evaluated.

**Results:**

Additive queries on PubMed retrieved 928 candidate papers. Of these, 376 were eligible based on abstract. Finally, 26 studies qualified for a full-text review, and 17 provided enough information for the study objectives. An additional 4 papers were added from the references of the reviewed papers. The results demonstrate massive variations in PPVs ranging from 8% to 83% according to the object of the decision support, with most results between 20% and 40%. The best results were observed when patients’ characteristics, such as comorbidity or laboratory test results, were taken into account. There was also an important variation in sensitivity, ranging from 38% to 91%.

**Conclusions:**

There is increasing reporting of alerts override in CPOE decision support. Several causes are discussed in the literature, the most important one being the clinical relevance of alerts. In this paper, we tried to assess formally the clinical relevance of alerts, using a near-strong proxy, which is the PPV of alerts, or any way to express it such as the rate of true and false positive alerts. In doing this literature review, three inferences were drawn. First, very few papers report direct or enough indirect elements that support the use or the computation of PPV, which is a gold standard for all diagnostic tools in medicine and should be systematically reported for decision support. Second, the PPV varies a lot according to the typology of decision support, so that overall rates are not useful, but must be reported by the type of alert. Finally, in general, the PPVs are below or near 50%, which can be considered as very low.

## Introduction

Computerized patient records and computerized provider order entry (CPOE) systems are recognized as major tools in efforts to improve the safety and efficiency of care. Computerized patient records are the cornerstone of information sharing among care providers, and increasingly with patients; they contribute to improving the continuum of care and patient safety. The way CPOE improves processes rests on 3 pillars. The first pillar is formal structured order entry, which improves both completeness and readability. The second embeds CPOE into complete care processes such as medication loops or clinical pathways. The third pillar is the decision support capability during the ordering process, such as the provision of extensive information on the drugs being prescribed or the links made between the current order and other elements of the patient’s record such as problems, laboratory results, and other drugs or diagnoses. Numerous studies have reported the positive effects of clinical decision support systems (CDSS) on patient outcomes such as fewer duplicate orders, dosage errors, drug interactions, and missed or delayed actions using reminders, to name a few [1-4]. The benefits of CPOE have already been demonstrated in the improved cost-efficiency of care, either directly, by lowering adverse events and duplicate orders, or indirectly, by reducing lengths of stay [5,6]. Nevertheless, the burden of alerts and reminders must not be too high or *alert fatigue* could cause clinicians to override both important and unimportant alerts, thus jeopardizing the improvements in safety that a CDSS should be expected to bring [7]. In other words, the CDSS’s specificity (Sp) must be high. A few studies have reported on the unintended effects of CDSS in CPOE [8-10] and their occasional dramatic consequences on patient safety. These were related to delays in reporting adverse events, and thus therapy, leading to specific infectious or thrombotic complications in treatment [11] or to the cancellation of QT interval-alert generation after proposed measures to reduce alert overload [12]. This is not a marginal problem. For example, a 2013 study published by Yeh, analyzing more than 1 million prescriptions from outpatient settings in Taiwan, reported a 91.5% override rate on the approximately 11,000 drug-drug interaction alerts proposed [13]. Understanding the reasons why clinicians override CDSS in CPOE has since received a lot of attention [14,15]. In recent years, numerous studies have been published on the topic of alert improvements for CPOE. These addressed the theoretical background, such as models and frameworks [16], data representation [17] or behavioral theories [18], usability and interfaces [19,20], perceptions and expectations [21], simulation [22], effectiveness monitoring [23,24], and decision support Sp [25], among other issues.

This study focuses on the predictive value of CPOE alerts. One can consider the CDSS in CPOE to be akin to any other decision support instrument in medicine: a tool with positive predictive values (PPVs) and negative predictive values (NPVs). As mentioned above, some previous studies have focused on evaluating the predictive value of decision support in CPOE, and the PPVs reported were usually below 20% and as low as 5% [26,27]. A study by van der Sijs et al stated that 49% to 96% of alerts were overridden [28] and identified a range of human factors responsible:

- alert fatigue due to a poor signal-to-noise ratio as a result of a low PPV
- usability issues such as bad ergonomics, misinterpretation, or unnoticed alerts
- disagreements with guidelines
- physicians’ belief in their own knowledge
- lack of time

Further understanding has been provided by questionnaires and focus groups that allowed physicians to evaluate the most important factors for useful, easy-to-use alerts [29,30]. These showed that drug-related alerts were rated more useful than alerts reminding the clinician of the state of the patient’s health or disease. Shah et al suggested that an approach based on a careful selection of alerts so as to improve the relevancy, severity, likelihood, and strength of clinical evidence would improve the acceptance of alerts [31]. Bates et al put forward “Ten commandments for effective clinical decision support” such as speed of the information system, anticipation of clinician needs and provide information to clinicians at the time they need it, integration suggestions with practice, offer an alternative, change of direction rather than stop or management, and maintenance of knowledge-based systems [32].

As stated, most alerts are overridden. Although numerous authors speak about the number of alerts, or the pertinence of alerts, we have been interested in trying to assess clearly the PPV of alerts, and thus the rate of true and false positive alerts. In doing this review, three inferences were drawn. First, very few papers report direct or enough indirect elements that support the use or the computation of PPV, which is a gold standard for all diagnostic tools in medicine, which is why it should be systematically reported for decision support. Second, the PPV varies a lot according to the typology of decision support and would have to be reported by the type of alert. Third is that, in general, the PPVs can be considered as very low—below 50% or near 50%.

Due to the high expectations health care professionals have for CDSS in CPOE, as well as the related costs and potential unintended consequences, we decided to carry out a systematic review of the literature on CPOE, CDSS, and predictive value, and their associations with alert fatigue. We start from the assumption that a low PPV would explain why majority of alerts are overridden. We framed this systematic review to determine the real PPV of CPOE alerts.

## Methods

### Selection Criteria

We targeted publications evaluating clinically relevant alert in computerized patient records implementing CPOE.

### Search Strategy

A search of the literature was made using PubMed for work published between February 2009 and March 2015, using the following queries: (CPOE[all fields] OR “Medical Order Entry Systems”[all fields] OR “Alert Systems”[all fields] OR “Order Entry”[all fields] OR “Decision support Systems”[all fields]) AND (sensitivity[All Fields] OR sensibility[All Fields] OR predictive[All Fields]) OR (fatigue[All Fields] OR overload[All Fields] OR overcharge[All Fields] OR burden[All Fields] OR override[All Fields] OR overalerting[All Fields] OR ignore[All Fields]). The following meanings were searched for *decision support*: CPOE, medical order entry systems, alert systems, order entry, and decision support systems. The following meanings were searched for *relevance*: sensitivity, sensibility, predictive, fatigue, overload, overcharge, burden, override, over alerting, and ignore.

The following limits were applied to all queries: English language, only papers available in full text, assessing medication, and numerical data available.

We excluded qualitative studies, user-satisfaction or opinion surveys, physician adherence studies, and analyses of the impact of human factors.

### Selection of Relevant Publications

First, the 3 reviewers (DC, GF, and CL) selected references independently based on their titles and according to the review study’s inclusion and exclusion criteria. When results were discordant, the final choice was made by consensus. Next, they independently read and assessed the abstracts of all the papers identified. When no abstract was available, full-text papers were retrieved and reviewed so that only relevant papers were retained. Again, the 3 reviewers solved any disagreements by consensus. In the absence of an agreement, the abstract was provisionally included for consideration subject to reading the full text.

Abstracts that were rated as relevant to the research question were kept, and all full-text papers were retrieved. Then, each retrieved paper’s reference section was searched for additional relevant literature that might be included.

Of the reviewers, 2 (DC and GF) assessed the quality of the papers selected by using a standardized evaluation process based on the exclusion and inclusion criteria. For papers to be selected for the final review, the levels of predictive value, sensitivity (Se; ability to generate alerts in potentially dangerous situations), or Sp (inability to prevent irrelevant alerts) were retrieved or calculated if possible. Se was defined as the number of patients with an adverse drug event (ADE) detected by an alert, out of the total number of patients with a positive ADE. Sp was defined as the number of patients without an ADE and with no warning alert, out of the total number of patients without an ADE. The PPV was defined as the number of relevant medication alerts (true positives) out of the total number of alerts (sum of true and false positives). Evaluation disagreements between the 2 reviewers were resolved by the third reviewer (CL).

## Results

### Selection of Studies

**Figure 1:**
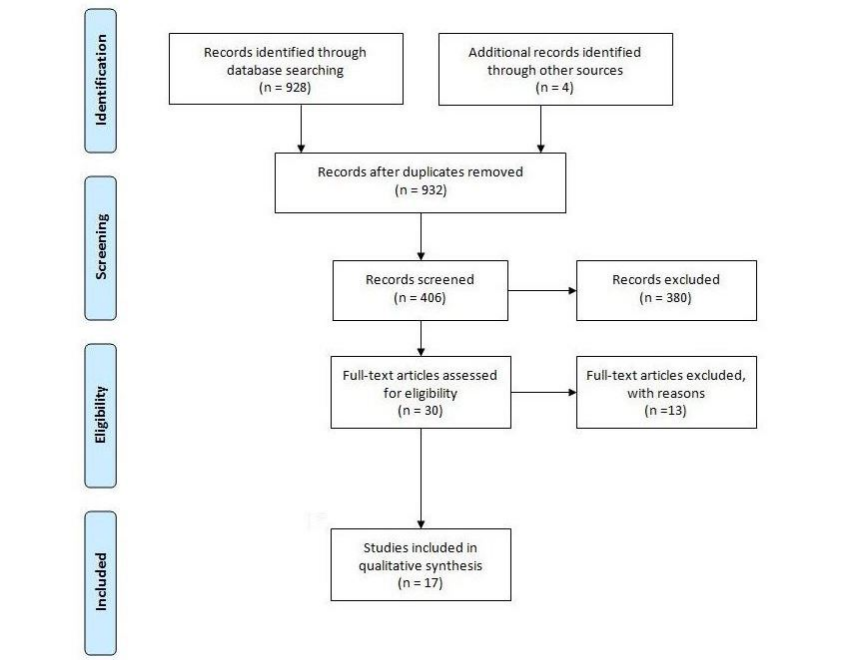
Flowchart describing the systematic literature review process.

The database search retrieved 928 matching references. A first evaluation based on MEDLINE summary allowed identifying 402 potentially interesting papers. Then, a second deeper analysis based on abstracts and applying the inclusion and exclusion criteria resulted in the exclusion of 311 articles, thus reducing the initial set to 91 reports. Out of these, 26 full-text papers were retrieved, reviewed, and included in the next phase of the review. The additional search through the selected studies’ reference sections resulted in 20 additional potentially relevant papers. Of these, 4 were included in our analysis. The review selection process is summarized in Figure 1.

### Description of Studies

Including the additional search references, the final sample of 17 studies that met our eligibility criteria, as listed in Table 1, were published between 1998 and 2015. The papers predominantly analyzed interruptive alerts (n=7/8 notified). Various alert targets were used and are described in Table 2. The main ones described were drug-lab interactions (n=11), drug-dosage interactions (n=8), drug-drug interactions (n=6), duplicate orders (n=3), and drug–allergy interactions (n=3).

These papers report the predictive value or Se and Sp of the alerts studied. As shown in Table 3, four papers did not report any PPV, although this study’s authors were able to calculate it for two of those papers. The PPV found in the papers were usually low and heterogeneous, mostly between 20% and 40%. Despite the diversity of target alerts, alert notifications, study designs, and study periods of the papers included in this review, it seems that PPVs were higher for drug-lab interactions (2.3%-83%) than they were for drug-dosage interactions (8%-13.8%), or drug-drug interactions (1.6%-48%). Furthermore, advanced CDSS [49] showed higher PPV than the more basic ones (17%-97%).

#### The Types of Alert Influencing PPV

In general, PPV increased when the risk increased. For example, PPV was higher for drug-dosage interactions than for drug-lab interactions. This is probably because of the higher risk of experiencing an ADE [48]. Furthermore, the PPV was lower in prevention (the opportunity to prevent ADEs) than in detection (evaluate or treat possible existing ADEs): 24% versus 97% [46]. Indeed PPV is related to the prevalence (Prev) unlike Se and Sp, which are only related to the test as defined as defined as follows: PPV=(Se×Prev)÷(Se×Prev+(1−Sp)×(1−Prev)). Therefore, in prevention settings, the prevalence of disease is likely to be very low, so the PPV will also therefore be low. Additionally, it was shown that the PPV of alerts targeting drug-lab interactions varied with the choice of the alarm signal. Indeed, for a laboratory value lower than the maximum defined value, the PPV of the alert was 36% (95% CI 29-43). If an alert was activated after at least a 50% decrease in the value between the last two laboratory results, the PPV increased to 83% (95% CI 62-104). For two consecutive decreases, with at least a 25% difference between the third most recent and the most recent platelet count, the PPV was 40% (95% CI 32-48) [50]. 

Furthermore, it has been shown that the PPV of safety alerts aimed at high-risk patients was higher (PPV=14%) than when dealing with initiation of a drug (PPV=6%), ongoing use of a drug (PPV=6%), advice (PPV=7%), and medication used to treat an ADE (PPV=0%) [28]. In summary, the PPV of alerts is usually very low. However, several factors seem to improve PPV.

#### Contextual Information Improves PPV

The PPV of advanced alerts is higher than for basic alerts because they are more specific. Advanced CDSS, such as using patients’ characteristics and laboratory test results, have a higher PPV than basic ones. For example, Eppenga et al showed that using information from the laboratory and a few other specific pieces of information increased the PPV from 12.2% to 23.3% (*P*<.05) and that PPV was higher in advanced systems than in basic ones (17% vs 5.8%, *P*<.05) [37]. Numerous factors can influence the PPV, mostly because they will have influence of the population considered for the alert. For example, not specifying the administration route can sometimes decrease the PPV, for example in some topical treatments. This is because the risk of developing an ADE can vary according to the administration route [50]. Further advances in dosing alert systems should aim to improve the Se of alerts. The Se of the system for identifying dosing errors increased from 54.1% (95% CI 47.8-60.3) to 60.3% (95% CI 54.0-66.3) in a customized dose range system (*P*=.02). The system’s Se for underdosage was 49.6% without customization, and this increased to 60.3% with customization (*P*=.01) [47]. Furthermore, it has been highlighted that PPV differs according to patients’ characteristics and comorbidity: for alerts on the risk of developing hypoglycemia, the PPV was higher for patients with sulfonylureas in their drug regimens (45.7% vs 28.4%, *P*=.04) and for patients with three or more chronic medical conditions (35.7% vs 22.7%, *P*=.049). The PPV of an alert warning of the risk of developing hyperkalemia was higher for patients with serum creatinine >2.0 mg/dL (50.0% vs 16.0%, *P*=.01) [38].

The PPV can vary according to the types of alerts. Among the 5 types of alerts with the best PPV (34.1%-73.3%), 3 were drug-lab interactions, which are advanced alerts. In parallel, of the 10 alerts described as being the least relevant (PPV between 0% and 4.5%), 8 were drug-drug interactions [37].

Finally, the PPV varies according to the specific goal. A study of alerts aimed at identifying 4 types of ADE showed that some of them could have a lower PPV: the PPV was only 4.0% (95% CI 1.3-9.1) for hypokalemia versus 31.2% (95% CI 18.2-46.6) for hypoglycemia, 31.1% (95% CI 25.1-37.8) for hyperkalemia, and 20.6% (95% CI 11.7-32.1) for thrombocytopenia. Furthermore, the effect of an alert can differ according to the medical specialty. In a study by Riggio et al, a surgery department ordered laboratory tests earlier than general medicine department when alerts were activated, probably because surgeons were more aware of the importance of the platelet counts that were being observed in the study [33]. The PPV can also vary according to the alert’s pharmacological target. For example, anti-infective drugs are excluded from alerts concerning drug dosage interactions to limit the number of false positives because these drugs could present patients specific dosing adjustment and multiple indications [44].

**Table 1:** Characteristics of the studies included in the paper.

Source	Study design	Study period	Study site	Specialty	Patient care	Number of patients with an alert
Riggio et al, 2008 [33]	Control and intervention	3 weeks	728-bed hospital	Medicine, surgery, pediatric	Inpatient	Control group: 47; intervention group: 53
Cash, 2009 [34]	Retrospective analysis	N/A^a^	Hospital	Pediatric	Inpatient	N/A
Van der Sijs et al, 2010 [35]	Control/intervention	1 month	807-bed hospital	N/A	Inpatient	N/A
FitzHenry et al, 2011 [36]	Retrospective analysis	7 months	807-bed hospital	N/A	Inpatient	2404
Eppenga et al, 2014 [37]	Cross-sectional	5 days	800-bed hospital	N/A	Inpatient	619
Moore et al, 2009 [38]	Prospective observational	5 months	684-bed hospital	N/A	N/A	456
Fritz et al, 2012 [39]	Prospective observational	N/A	850-bed hospital	Internal medicine	Inpatient	100
Harinstein et al, 2012 [40]	Prospective observational	8 weeks	Medical center	Medical and cardiac intensive care	Inpatient	64
Zorina et al, 2012 [41]	Cross-sectional	1 year	850-bed hospital	Neurological	Inpatient	484
Beeler et al, 2013 [42]	Retrospective analysis	90 weeks	850-bed hospital	N/A	Inpatient	922
Rommers et al, 2013 [27]	Prospective observational	5 months	Hospital	Internal medicine, cardiology, lung, gastrointestinal, hematology	Inpatient	931
Stultz et al, 2013 [43]	Retrospective analysis	1 month	350-bed hospital	Pediatric	Inpatient	573
Stultz et al, 2014 [44]	Retrospective analysis	1 month	350-bed hospital	Pediatric	Inpatient	189
Dormann et al, 2004 [45]	Prospective study/retrospective analysis	6 months	Hospital	Gastroenterological	N/A	377
Raschke et al, 1998 [46]	Prospective case	6 months	650-bed hospital	Nonobstetrics	N/A	9306
Silverman et al, 2004 [47]	Retrospective analysis	3 one-year periods	726-bed teaching institution	Tertiary care	N/A	N/A
Handler et al, 2007 [48]	Systematic review	12 studies	Hospital	N/A	N/A	N/A

^a^N/A: not applicable.

**Table 2:** Characteristics of alerts included in the paper.

Source	Alert notification	Alert origin	Alert target
Riggio et al, 2008	Interruptive alert	—	Drug-lab interaction: heparin-induced thrombocytopenia
Cash, 2009	Interruptive alert	—	Drug-drug interaction Drug-lab interaction Duplicate order Drug-dosage interaction Drug-allergy interaction
Van der Sijs et al, 2010	Interruptive alert	Commercial system	Drug-dosage interaction: overdosage Drug-drug interaction Drug–dosage interaction Drug-allergy interaction Drug-pregnancy interaction: contraindication Duplicate order Drug-lab interaction: bad renal function Drug-pharmacogenetic interaction: poor metabolizer
FitzHenry et al, 2011	Interruptive alert	—	Drug-dosage interaction: warfarin
Eppenga et al, 2014	Interruptive alert	—	Basic Drug-drug interaction Duplicate order
			Advanced Drug-drug interaction Drug-dosage interaction Drug-lab interaction Drug-lab interaction: missing laboratory value Drug-disease interaction Drug-age interaction
Moore et al, 2009	—	—	Drug-lab interaction developing adverse drug event (ADE): hypoglycemia, hypokalemia, hyperkalemia, and thrombocytopenia
Fritz et al, 2012	—	Commercial system	Drug-drug interaction
Harinstein et al, 2012	—	Commercial system	Drug-lab interaction: drug-induced thrombocytopenia
Zorina et al, 2012	—	Commercial system	Drug-drug interaction
Beeler et al, 2013	Noninterruptive alert	—	Drug-drug interaction
Rommers et al, 2013	—	—	Drug-lab interaction: ADE system
Stultz et al, 2013	Interruptive alert	—	Drug-dosage interaction
Stultz et al, 2014	Interruptive alert	—	Drug-dosage interaction
Dormann et al, 2003	—	—	Drug-lab interaction: predicted ADE
Raschke et al, 1998	—	—	Drug-monitoring interaction: predicted ADE Drug-age interaction: predicted ADE Drug-lab interaction: predicted ADE
Silverman et al, 2004	—	—	ADE detection system Drug-allergy interaction Drug-drug interaction Therapeutic duplication Drug-dosage interaction Drug-lab interaction
Handler et al, 2007	—	—	Antidote Drug-lab interaction Drug-dosage interaction: subtherapeutic medication levels

**Table 3:** Positive predictive value (PPV), sensitivity or specificty for studies included in the review.

Source	Number of alerts	Positive predictive value (%)	Sensitivity (%)	Specificity (%)	False positive (%)
Riggio et al, 2008	41,922	2.3	87	87	N/A^a^
Cash, 2009	—	1.4	N/A	N/A	N/A
Van der Sijs et al, 2010^a^	—	—	38-79 (n=29)	11-89 (n=19)	N/A
FitzHenry et al, 2011^b^	2308	—	N/A	N/A	46-85
Eppenga et al, 2014	Basic 2607/advanced 2256	Basic: 5.8 (n=150/2607)/advanced: 17 (*P*<.05)	N/A	N/A	N/A
Moore et al, 2009	611	4.0 (n=125)-31.2 (n=218)	N/A	N/A	N/A
Fritz et al, 2012	743	5.7 (n=3/53)-8 (n=29/362)	9.1(n=3/53) -87.9 (n=29/362)	N/A	N/A
Harinstein et al, 2012	350 (204/12/134)	36 (n=73/204)-83(n=10/12)	N/A	N/A	N/A
Zorina et al, 2012	1759/1082^c^	24/48^c^	70.6/72.4^c^	N/A	N/A
Beeler et al, 2013^d^	7902	1.6 (n=47/2866) (*P*=.002)	N/A	N/A	N/A
Rommers et al, 2013	2650 (963/722/437)	8 (n=204/2650)	N/A	N/A	N/A
Stultz et al, 2013^e^	3774	13.8	N/A	N/A	N/A
Stultz et al, 2014	257	Odds ratio (OR) 8 (95% CI 6.8-9.3)	OR 60.3 (95% CI 54.0-66.3) *P*=.02	OR 96.2 (95% CI: 96.0-96.3)	N/A
Dormann et al, 2003	2328 (1748/580)	Prospective study 25(n=574/2328)(13-40)/retrospective analysis 32(18-67)	91/40	23/76	N/A
Raschke et al, 1998	1116 (803/313)	24 (n=5/21)-97(n=190/196)	N/A	N/A	N/A
Silverman et al, 2004	3117/7390/6136	0-60	N/A	N/A	N/A
Handler et al, 2007	—	Antidotes: 9-11	N/A	N/A	N/A
	—	Laboratory test result: 3-27	N/A	N/A	N/A
		Supratherapeutic medication levels: 3-50	N/A	N/A	N/A

^a^N/A: not applicable.

^c^No PPV available.

^c^Values for two different programs of clinical decision support systems.

^d^Positive predictive value calculated for the review: PPV was defined as the quotient of the number of advice/interventions to prevent a possible adverse drug event and the total number of alerts generated.

^e^PPV calculated for the review: PPV was defined as number of correct alerts in comparison with Lexicomp.

## Discussion

### Principal Findings

The PPV found in the papers were rather low: 20% to 40%. Despite the heterogeneity of papers, it seems that several factors influence PPV. First, the PPV can vary with the types of alert such as the risk patients trying to be prevented. Furthermore, several factors seem to improve PPV such as contextual information. Indeed alerts that are more specific have a higher PPV than basic alerts specifying the administration route or patients’ characteristics for example. Moreover, PPV can differ according to alert’s pharmacological target or medical specialty.

Even the most basic systems usually show good Se. They thereby allow medical professionals to detect drug-related problems more comprehensively: a pharmacy department increased the number of its clinical interventions by 15% after the introduction of a CDSS [47]. However, the impact of a true positive alert can be paradoxical. For example, patients presented no reduction in ADEs, time to therapeutic intervention, or time to laboratory testing in an alert group, and physicians waited 1.6 days longer before stopping a treatment inducing ADE in that alert group (*P*=.049) [49]. This result could be because of alert fatigue induced by a low PPV.

This study has several limitations. First of all, we conducted our research using only PubMed, and carried no queries using EMBASE, Web of Science, or conference proceedings. The results are based on few reports, as only few studies reported all characteristics required to assess properly the contexts of decision support and their associated predictive values. There was a wide heterogeneity in how results were reported, completeness, and evaluation methodologies, thus limiting the reliability of pooling the PPV of alerts across publications. Because PPV varies with prevalence, the patient context, including population, hospital settings, and the like, has influence, and could not be considered. Thus, these results introduce some types of biases into the overall assessment.

Studies about interruptive alerts had some homogeneity in their methodology, and studies on decision support were mostly about 3 types interactions: drug-lab, drug-drug, and drug-dosage. These 3 types of interactions were the easiest to implement, and there are several large databases available for each of them. In general, systems that do not take patients’ specific clinical information into account and use only external databases demonstrate the lowest PPV; systems that have a specific source of knowledge and use the greatest number of patients’ individual characteristics have the highest PPV.

### Conclusions

The PPV of clinical decision support systems for CPOE, as reported in the literature, varies massively, from 5.8% to 83%, with the majority of results between 20% and 40%. Drug-drug interaction alerts have the lowest PPV, and drug-lab alerts have the highest.

Our literature review leads us to suggest that the best strategy to use with a CPOE is to adapt and carefully optimize the database driving the knowledge for activating alerts. Furthermore, the CDSS should take into account as many of the patient’s characteristics as possible. The efficiency of the alerts, and thus their PPV, is more important than a very large database of knowledge that may generate lots of false positives, which reduce PPV and generate alert fatigue.

Advanced alert systems should aim to improve PPV of alerts, while keeping a good Se. To reduce the number of false positive alerts, contextual data from different sources, such as the pharmacy, demographic data, or laboratory tests, should be integrated into the system.

The US Institute of Medicine has suggested that systems should be designed so as to make it “hard for people to do the wrong thing and easy for people to do the right thing” [51]. However, with PPVs as low as those seen in the literature, it seems, unfortunately, that many computerized patient records tend to make it hard for people to do the right thing and easy for people to do the wrong thing.

## Abbreviations

ADE: adverse drug event
CDSS: clinical decision support systems
CPOE: computerized provider order entry
PPV: positive predictive value
Se: Sensitivity
Sp: Specificity
